# Talin1 Mediates Tumor-Nerve Interactions in Prostate and Breast Cancer Cells

**DOI:** 10.7150/jca.127292

**Published:** 2026-01-08

**Authors:** Bor-Jang Hwang, Gloria Polanco, Sanam Sane, Igor C. Almeida, Kensei Tsuzaka, Frank Denaro, Khosrow Rezvani, Valerie A. Odero-Marah

**Affiliations:** 1 Center for Urban Health Disparities Research and Innovation, Department of Biology, Morgan State University, Baltimore, MD, USA.; 2 Department of Molecular Microbiology, Washington University School of Medicine, St. Louis, Missouri, USA.; 3 Biomedical and Translational Sciences, Sanford School of Medicine, University of South Dakota, Vermillion, SD, USA.; 4 Department of Biological Sciences, University of Texas El Paso, El Paso, TX, USA.; 5 Kaytee Bio, Co. & Ltd., Chiba, Japan.

**Keywords:** Talin1, Snail, exosome, neurite outgrowth, mH4

## Abstract

Prostate cancer (PCa) and breast cancer (BCa) are the leading causes of death in men and women in the US. Neurite outgrowth is a fundamental process in differentiating neurons and contributes to cancer progression. Snail transcription factor promotes cancer progression and regulates neurite outgrowth in PCa cells, but their molecular mechanisms are not fully understood. We hypothesize that Snail can stimulate neurite outgrowth through the secretion of extracellular vesicles. To test this hypothesis, we isolated exosomes from PCa (C4-2 non-silencing (NS) control and C4-2 Snail knockdown) and BCa (MCF7 Neo control and MCF7 Snail overexpressing) cells, which were confirmed by western blot analysis and Transmission Electron Microscopy. Proteomics of isolated exosomes from Snail-expressing C4-2 cancer cells shows predominantly Talin1 proteolyzed C-terminal rod domain and N-terminal head domain within exosomes, while full-length Talin1 is found in whole cell lysates. A significantly higher percentage of NPC (Neural Progenitor Cells) with neurite outgrowth is observed when cultured with conditioned medium or exosomes collected from C4-2 NS PCa or MCF7 Snail BCa cells expressing high levels of Snail compared to C4-2 Snail knockdown or MCF7 Neo, respectively. A similar trend is observed for increased average neurite length due to Snail expression. Furthermore, we find that mH_4_, a specific inhibitor of proteolyzed Talin1, reduces Snail-induced neurite outgrowth and AKT activation within neurons. Overall, Snail may promote cancer-nerve interactions *via* Talin1, indicating that Talin1 inhibitors can be a potent targeted therapy in malignant tumors with neurite outgrowth.

## Introduction

The nervous system communicates between the brain and the rest of the human body. In addition, recent research indicates active interactions between cancer cells and nerves infiltrating a variety of tumors. This type of tumor innervation in different types of cancer was summarized in a recent review 1. As a result, the nervous, vascular, and immune systems are utilized by tumors to favor cancer progression 2. Neurite outgrowth is when nerve cells assemble into networks by growing axons and dendrites during development. Since nerves travel through holes in the prostate capsule, PCa metastasis may not require lymphatic or vascular invasion. In 2008, Gustavo observed increased nerve density in PCa samples and cancer cells interacting with nerves in a perineural invasion, confirming neurite outgrowth in PCa 3. A suggested mechanism was noted with the nerve growth factor (NGF)/ NGF receptor signaling induced by PCa, observed to drive tumor neurogenesis and nerve infiltration in tumors 4. In BCa, more sensory nerves are found in Triple-Negative Breast Cancer (TNBC) invasive ductal carcinoma samples when stained with pan-neuronal marker β3-tubulin^5^. In the mouse model, dorsal root ganglia (DRG) increase metastasis when co-injected with MDA-MB-231 into mice and increase cell migration when co-cultured with MDA-MB-231 5. Another study using a sensory neuronal cell line 50B11 cultured in a conditioned medium MDA-MB-231 and MCF7 showed induced neurite growth 6. It appears that both neurotransmitters and growth factors are secreted from nerve cells promote cancer cell growth and angiogenesis in BCa 7.

Snail is a master gene that can regulate the epithelial-mesenchymal transition (EMT) process; by binding to several E-boxes in the promoter region, Snail down-regulates E-cadherin, thereby inducing EMT 8. As observed in the TCGA-PRAD database, high levels of Snail mRNA have been significantly associated with reduced disease-free survival (p = 0.001, p = 0.005, and p = 0.015) 9. Snail expression also serves as a PCa independent predictor of Gleason score, pathological stage, and disease recurrence, albeit with borderline significance (HR 1.8, 95% CI 1.0-3.5, p = 0.088) 9. EMT has been characterized in epithelial cancers in which tumor cells at the invasive front undergo this transition to promote invasion, migration, and subsequent metastasis in thyroid cancer, BCa, PCa, melanoma, and lung cancer 10, 11. Converging evidence indicates that Snail expression is associated with metastatic prostate patient tissue 12. We have shown that silencing the Snail protein reduces cell proliferation, migration, and invasion in C4-2 PCa cells C4-2 13. We also observed that cancer cells with higher Snail expression migrated and adhered more toward sNF96 or NS20Y nerve cells. In addition to the changes in cancer cell behavior, we also observed increased neurite outgrowth of PC12 and NS20Y nerve cells when co-cultured with conditioned medium from LNCaP cells overexpressing Snail or C4-2 non-silencing cells compared to LNCaP Neo control or C4-2 Snail knockdown cells, respectively 13. However, the mechanism(s) of action are unclear.

Talin1, a member of the Talin family, plays a key role in focal adhesions and can regulate cell behavior 14. As an intracellular adaptor, Talin1 connects integrins to the actin cytoskeleton, facilitating integrin activation 15-17. Talin1 is a 270 kDa protein consisting of a 47 kDa N-terminal head domain and a 220 kDa C-terminal rod domain. Cleavage by calpain can release both domains to increase binding of the Talin1 47 kDa head domain to FAK and β integrins and increase binding of the Talin1 220 kDa rod domain to β integrins, vinculin, and F-actin 18, 19. Cathepsin H (CtsH)-mediated processing of Talin1 has been observed in metastatic PC-3 prostate cancer cell lines, where increased CtsH expression is co-localized with Talin1 in focal adhesions 20. Significantly elevated levels of Talin1 have been detected in metastatic prostate tumors compared to primary tumors in human PCa specimens 21, 22. A high level of Talin1 was detected in TNBC samples and correlated with metastasis and worse prognosis 23. Interestingly, Talin1-dependent stimulation of axon regeneration and neurite outgrowth has been reported in nerve cells 24.

Extracellular vesicles, including microvesicles and exosomes, carry unique protein and RNA cargo, facilitating cell-cell communication and other physiological processes 25, 26. Exosomes are lipid membrane bilayer-encapsulated vesicles secreted by cells into the extracellular space with various sizes between 30-200 nm and a density of 1.13-1.19 g/mL 25, 27, 28. They carry many biologically active materials such as DNAs, RNAs, proteins, and lipids that play pivotal roles in intercellular communication. Studies indicate that exosomes from cancer stem cells support PCa tumorigenesis by promoting angiogenesis. 29, 30. Recent research suggests that exosomes from the tumor microenvironment significantly regulate prostate cell survival, proliferation, angiogenesis, immune evasion 31, and neurite outgrowth on PC12 cells 32.

This study aims to elucidate the molecular mechanisms of cancer-neuronal cell interactions, which have not been well-studied.

## Materials and Methods

### Reagents

RPMI, DMEM media, Matrigel and penicillin/streptomycin were purchased from (Corning Cellgro, New York, NY.). Fetal Bovine Serum (FBS), 10X PBS, Neurite outgrowth staining kit and exosome-depleted serum were purchased from Fisher Scientific, Waltham, MA. Mouse monoclonal anti-Snail, rabbit monoclonal anti-Talin1, and rabbit monoclonal anti-P-AKT (S473 and T308), were from Cell Signaling Technology, Danvers, MA. AKT, and β-actin antibodies were purchased from Santa Cruz Biotechnology, Dallas, TX. Secondary HRP-conjugated anti-mouse and anti-rabbit antibodies were purchased from Millipore-Sigma, Burlington, MA. G418 (aminoglycoside antibiotic) was purchased from Gemini Bio, West Sacramento, CA. Luminata Forte ECL reagent and the protease inhibitor cocktail were from Roche Molecular Biochemicals, Indianapolis, IN. Rat tail collagen I was from BD Biosciences (Franklin Lakes, NJ). DMEM/F12, B27, N2, Laminin FGF2 and accutase are from Gibco-Peprotech-ThermoFisher, Waltham, MA. Neural Progenitor Cells (ACS-5004), NPC Growth Medium (ATCC ACS-3003), DMEM: F12 (ATCC 30-2006) and Cell Basement Membrane (ACS-3035) were purchased from American Tissue Cell Culture (ATCC), Manassas, VA. Talin1 inhibitor, mH4 and F3 protease inhibitor control, was obtained from Dr. Kensei Tsuzaka, Kaytee Bio, Co. & Ltd., Chiba, Japan. mH4 was dissolved in DMSO to a stock solution of 14 mg/ml. Rheumatoid arthritis (RA) patient plasma was obtained from Tokyo Dental College Ichikawa General Hospital, Chiba, Japan. All methods using human samples were carried out in accordance with relevant guidelines and regulations. All experimental protocols using human samples were approved by the Ethics Committee of Tokyo Dental College Ichikawa General Hospital. Informed consent was obtained from all subjects or their legal guardians.

### Cell culture

RWPE-1 were derived from normal adult human prostate tissue obtained from a 54-year-old White male and were transfected with a single copy of human papillomavirus 18 (HPV-18); these cells have a reported doubling time of approximately (~) 5 days 33. DU145 cells were derived from a brain metastasis line and have a doubling time of ~ 34 hours. LNCaP cells, derived from a lymph node metastasis, and 22RV1 cells, derived from the CWR22R xenograft derivative, have reported doubling times of ~2-3 days. PC3 cells, derived from a lumbar vertebral metastasis, and C4-2, derived from a vertebral metastasis of an LNCaP cell xenograft in mice, are considered more aggressive with doubling times of 1-2 days. MDA-PCA-2b cells were derived from a 63-year-old black male with a doubling time of 42-73 hours. MCF10A cells were derived from benign proliferative breast tissue and spontaneously immortalized without defined factors from a 36-year-old white female. It has a doubling time of 48 hours 34. BT474 cells were derived from a 60-year-old triple-positive (ER, PR, and HER2) White patient and have a doubling time of 3.5 days (Cellosaurus data bank). T47D cells were derived from a HER2-negative patient and have a doubling time of ~36 hours 35. MDA-MB-231 and BT549 cells were derived from TNBC patients. MDA-MB-231 is an adenocarcinoma cell line with a short doubling time of ~ 20 hours, whereas BT549 is a ductal carcinoma cell line that proliferates more slowly, with a doubling time of approximately 3.7 days (Cellosaurus data bank). MCF7 is an adenocarcinoma cell line derived from a 69-year-old HER2-negative White female and has a doubling time of ~ 24 hours 36. All breast cancer cell lines, along with rat pheochromocytoma PC-12 nerve cells, were obtained from ATCC, Manassas, VA. C4-2 non-silencing (NS) and C4-2 E8 stable Snail knockdown cells were previously generated using shRNA^37^. LNCaP/MCF7-Neo and LNCaP/MCF7-Snail overexpression cell lines were previously generated in our laboratory by stably transfection with pcDNA-3.1-Neo vector (control) and pcDNA-3.1-Snail cDNA, respectively 33.

All PCa and BCa cells were maintained in RPMI-1640, 100 IU Penicillin and 100 μg/ml Streptomycin, and 10% fetal bovine serum. PC-12 was cultured in RPMI-1640 containing 1X Penicillin/Streptomycin, 5% fetal bovine serum, and 10% horse serum. Human neural progenitor cell (NPC) was obtained from ATCC, Manassas, VA. NPC cells were grown in DMEM: F12 (ATCC 30-2006), mixed with NPC Growth Medium (ATCC ACS-3003) and Pen/Strep (100 U/ml) (Gibco-15140122). Cells were detached with Accutase (Gibco-A1110501) for ~ 5 min. After neutralization with medium and centrifugation at 200xg for 5 min, cells were then seeded in plates previously coated with Cell Basement Membrane (ACS-3035) in DMEM/F12.

### Conditioned media collection

2×10^6^ Cells (C4-2 NS/Snail shRNA and MCF7-Neo/Snail, and) were plated in p100 dishes in complete RPMI media overnight. Subsequently, the media was removed and phenol-red free RPMI media containing 10% exosome-depleted (Exo-D) serum was added and incubated at 37 ºC for 48 h. After 48 h, the supernatant was collected, spun down for 5 min, and utilized as conditioned medium for experiments.

### Neurite outgrowth assay

Briefly, for the quantitative neurite outgrowth assay, PC12 nerve cells (6 ×10^3^ cells/well) were plated in 96-well dishes (100 μl) overnight. After attachment, the media was removed and conditioned medium (100 μl) derived from PCa or BCa cells was added, or control medium or exosome-depleted medium. For Talin1 inhibitor treatments, we utilized 280 μg/ml mH4 or 2% DMSO for the control. Cell images were captured using a Nikon Eclipse TE2000-S Inverted Fluorescence Microscope two days after the co-culture. The experiments with mH4 Talin1 inhibitor in NPCs were further stained/fixed with 0.5% crystal violet in 20% methanol to enhance the cell image for further quantification after the 2-day co-culture. NPC images were taken by a Moticam 400 microscopic imaging system. Images from both PC12 and NPC experiments were analyzed using ImageJ Fiji Just software.

### Proteomic analysis

The proteomic analysis was performed in a similar manner as described in Kleber Silva Ribeiro's work on extracellular vesicles released by two Trypanosoma cruzi 38. In brief, extracellular vesicle (EV) populations were enriched from cell supernatant of C4-2NS (control) or C4-2 E8 (Snail knockdown) by differential ultracentrifugation at 300, 3000, and 10,000xg. The samples were reduced in 10 mM dithiothreitol at 56°C for 1h. Samples were then alkylated by adding in 50mM iodoacetamide at room temperature in the dark. Extracellular vesicles were diluted to 3 volumes by adding 100 mM ammonium acetate and digested with proteomics-grade trypsin (Sigma-Aldrich) at 1:50 ratio of enzyme:sample for 16 h at 37 °C. To stop the reaction, 2 µL of glacial acetic acid was added. Digested samples were concentrated in Speedvac (1-2 h), resuspended in 400 µL 0.1% trifluoroacetic acid (TFA), and desalted using Sep-Pak TC18 Light silica-based bonded phase columns (Millipore). The columns were pre-conditioned with 2 mL methanol, 2 mL of 0.1% TFA in 50% acetonitrile (ACN), then 2 mL 0.1% TFA before loading the samples. Then, the columns were washed with 4 mL 0.1% TFA and eluted with 2 mL of 0.1% TFA in 50% ACN. Samples were dried to ~50 µL volume and subjected to high-resolution LC-MS/MS analysis in a QE Orbitrap (Thermo Fisher Scientific).

### Talin1 IP followed by IB

Immunoprecipitation (IP) experiments were conducted as previously described using magnetic protein G beads with immobilized antibodies mixed per the manufacturer's instructions (Thermo Fisher Scientific) 39. Yield proteins were subjected to 4-20% gradient Sodium Dodecyl Sulfate-Polyacrylamide Gel Electrophoresis (SDS/PAGE) followed by western blot using the appropriate antibodies.

### Exosome isolation

Conditioned medium (CM) was collected as previously described and centrifuged at 2000 × g for 30 minutes to remove cells and debris. 100 ml of conditioned medium was concentrated to 10 ml, followed by buffer exchange to PBS (phosphate-buffered saline) using Amicon^®^ Ultra-2ml (30K, SigmaMillipore, Darmstadt, Germany). The concentrated solutions were then mixed with exosome isolation reagent (Invitrogen Total Exosome Isolation Reagent, Thermo Fisher Scientific) at 2:1 ratio and incubated at 4 ºC overnight. The mixtures were then centrifuged at 10,000 × g for 1 hour at 4 °C. The pelleted exosomes were resuspended in PBS and stored at -80 ºC.

### Transmission Electron Microscopy (TEM) for exosomes

Exosome pellets were fixed in 2% paraformaldehyde/PBS for at least 4 hours (O/N) and then 100 μl of 4% Osmium tetroxide at room temperature for 45 min. The pellets were then washed with PBS 2 times and resuspended in 100 μl PBS. The exosome solutions were applied on top of Lacey Carbon grids for at least 15 minutes, and cut filter paper tips were subsequently used to absorb excess liquid, followed by air drying. The grids were stored in the Drierite-containing chamber prior to TEM imaging. The TEM images were taken with a JEM-1400 Transmission Electron Microscope at the Core facility of Morgan State University.

### Western blot analysis

Cell lysates were collected using lysis buffer (1X modified RIPA buffer, 1X Protease inhibitor, 1 mM PMSF, and 1 mM sodium orthovanadate). Supernatants were collected and quantified using a BCA assay (Thermo Scientific, Waltham, MA). 30 μg of cell lysate was resolved using 8%, 12%, or 16 % (based on the protein sizes of interest) sodium dodecyl sulfate-polyacrylamide (SDS) gel electrophoresis, followed by electro-transferring onto nitrocellulose membranes (Bio-Rad Laboratories, Hercules, CA). Membranes were incubated with primary antibody and HRP-conjugated secondary mouse or rabbit antibody, followed by visualization using Luminata Forte ECL reagent in an Amersham TM imager 680. The blots were subsequently stripped using Restore Western blot stripping buffer (Pierce Biotechnology, Rockford, IL) and re-probed with β-actin antibodies for endogenous control.

### Flow-cytometry analysis

To determine the regulation of AKT phosphorylation at Ser473 and Thr308 (p-AKT (S473) and P-AKT (T308)), we used a BD Accuri™ C6 plus cell cytometer with 488nm and 640nm excitation lasers. All flow cytometry analysis was conducted according to the manufacturer's standard protocols (BD Biosciences, San Jose, CA). Treated C4-2 NS control cells with non-silencing shRNA, and C4-2 cells with stable shRNA Snail knockdown (C4-2 E8) were analyzed by flow for expression of p-AKT (S473) and pAKT (T308) following respective antibody staining protocols provided by the manufacturer (BD Biosciences). Data was analyzed using FlowJo v10.8 (BD Biosciences).

### Statistical analysis

We used one-way ANOVA followed by Tukey post hoc tests or the Holm-Šídák test using GraphPad Prism 9 (La Jolla, CA, USA) when appropriate. We had at least 6 individual measured microscopic fields per treated group to statistically compare neuronal length. The means were compared considering a P value of ≤ 0.05 as a significant difference (mean ± SD) (*P < 0.05, **P < 0.01, ***P < 0.001 and **** P < 0.0001).

## Results

### High Snail protein expression is observed in more aggressive PCa and BCa cell lines

**Figure 1:**
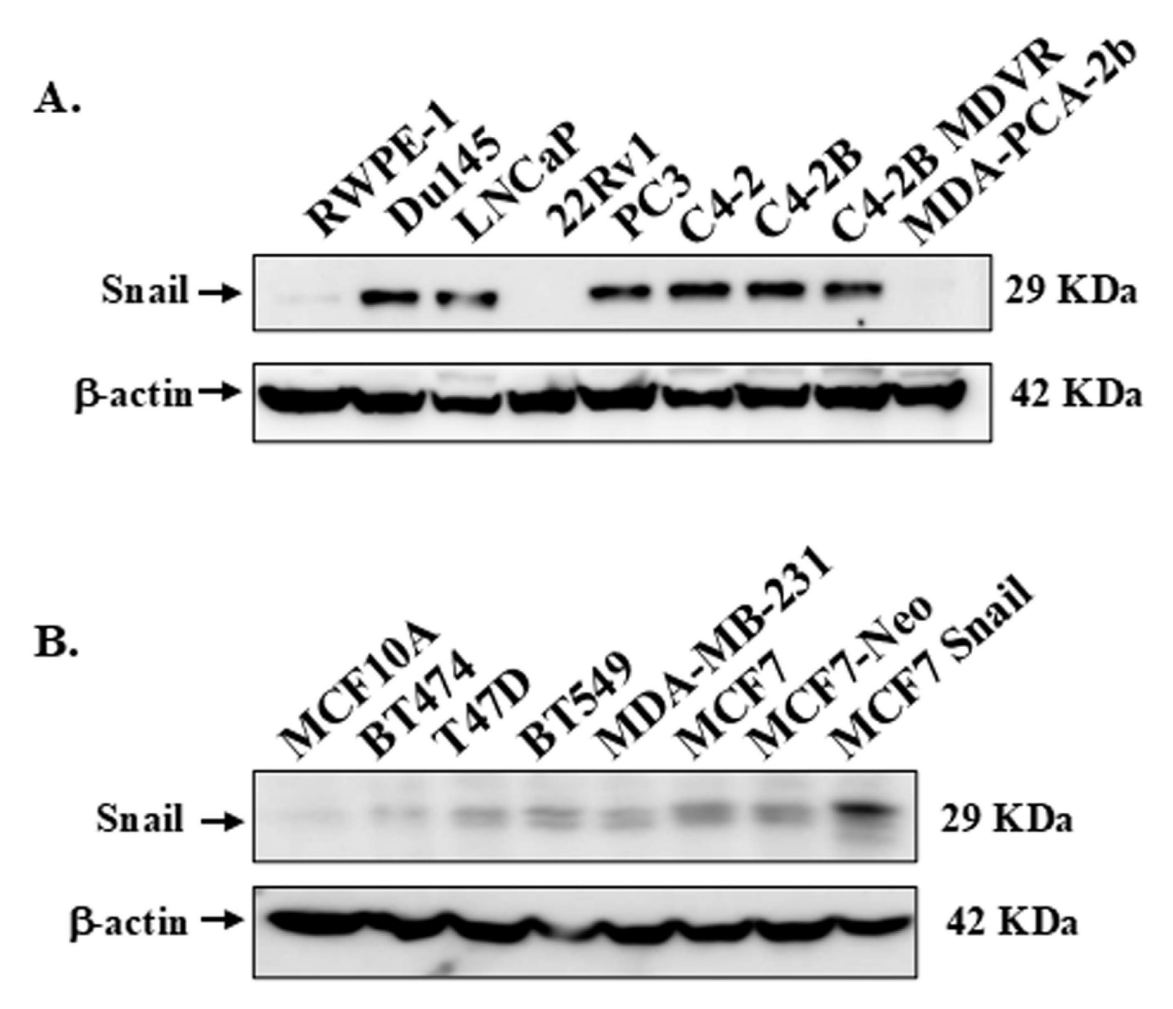
** Expression of Snail in PCa and BCa cell lines.** Snail expression was analyzed by western blot analysis in a panel of (A) PCa cell lines and (B) BCa cell lines. β-Actin was utilized as a loading control. The result is representative of at least 3 independent experiments.

First, we examined Snail expression in various PCa and BCa cell lines to be utilized by Western blots analysis. The information on PCa and BCa cells utilized, including the doubling times, is summarized in Table S1. Generally, although not universally, higher levels of Snail protein were observed in more aggressive cancer cell lines (Figure 1A, B). In Figure 1A, high levels of Snail protein were detected in the more aggressive cell lines: Du145, PC3, C4-2, and C4-2 derived lines (C4-2B, C4-2B MDVR) that originated from either brain or bone metastasis, while LNCaP cells had moderate levels of Snail. Normal RWPE-1, 22Rv1 and MDA-PCA-2b cells had low levels of Snail expression (Figure 1A). Additionally, the BCa cell lines expressed higher Snail levels compared to normal epithelial MCF10A cells (Figure 1B).

### PC12 rat nerve cells display increased neurite outgrowth when co-cultured with conditioned medium from BCa and PCa cells expressing higher Snail protein levels

**Figure 2:**
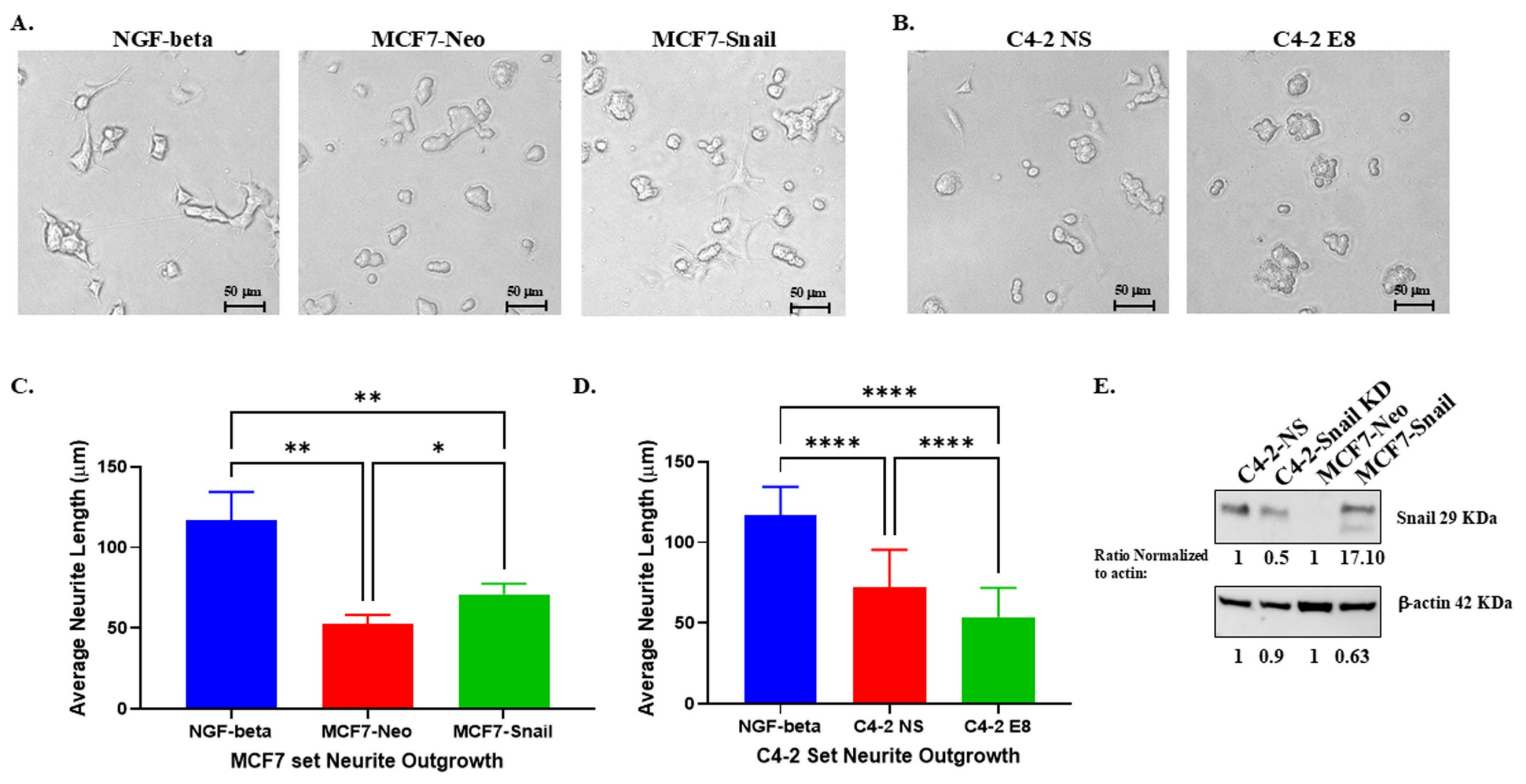
** PC12 rat neuronal cells show higher neurite outgrowth in response to conditioned media from cancer cells with higher Snail expression.** PC12 cells were grown in exosome-depleted medium or condition media (CM) collected from (A) MCF7 Neo control or Snail-overexpressing cells grown in exosome-depleted media, or (B) C4-2 non-silencing control (C4-2 NS) or C4-2 with stable Snail knockdown (C4-2 E8) for 4 days. Cells were then fixed in 4% formaldehyde in PBS, and images were taken with a Nikon Eclipse TE2000-S Inverted Fluorescence Microscope. Average neuron length of the brightfield images was measured and analyzed with ImageJ Fiji IS Just software for PC12 cells cultured with (C) MCF7 Neo/Snail CM or (D) C4-2 NS/E8 CM. (E) Snail expression in C4-2 NS/E8 and MCF7 Neo/Snail was analyzed by Western blot analysis. Quantification of Snail was performed using Image J software and normalized to β-Actin, which was utilized as a loading control. Statistical analyses were done using GraphPad Prism software (****p< 0.0001, **p < 0.01, *p < 0.05). Bars represent the SD of the mean. Results are representative of 3 experiments performed in triplicate.

In our previous study 13, we demonstrated that Snail in PCa cells promotes neurite outgrowth in PC12 and NS20Y nerve cells. For our current investigation, we collected conditioned medium (CM) from BCa MCF7-Neo and MCF7-Snail overexpression cells; PCa C4-2 non-silencing (NS) and C4-2 Snail knockdown (E8) cells to determine the response of PC12 neuronal cells to the CM of cancer cell lines in the presence and the absence of Snail proteins. The results revealed that Snail overexpression increased neurite outgrowth in PC12 cells, while the knockdown of Snail in C4-2 prostate cells reduced the outgrowth of PC12 nerve cells compared to Neo and NS controls, respectively, which could be observed both morphologically (Figure 2A and 2B). Quantification of the neurite outgrowth length confirmed that Snail overexpression significantly increased average neurite length in BCa cells, while Snail knockdown in PCa cells did the converse (Figure 2C and 2D). We further confirmed that Snail is higher in MCF7-Snail cells compared to MCF7-Neo and that Snail expression is lower in C4-2 E8 cells with Snail knockdown compared to C4-2 NS control cells (Figure 2E). These sets of gain-of-function (MCF-7-Neo) and loss-of-function (C4-2) results indicate that Snail has a dominant impact on the neurite outgrowth in these two cancer cell lines.

### Extracellular vesicles from Snail-expressing PCa cells contain Talin1

**Figure 3:**
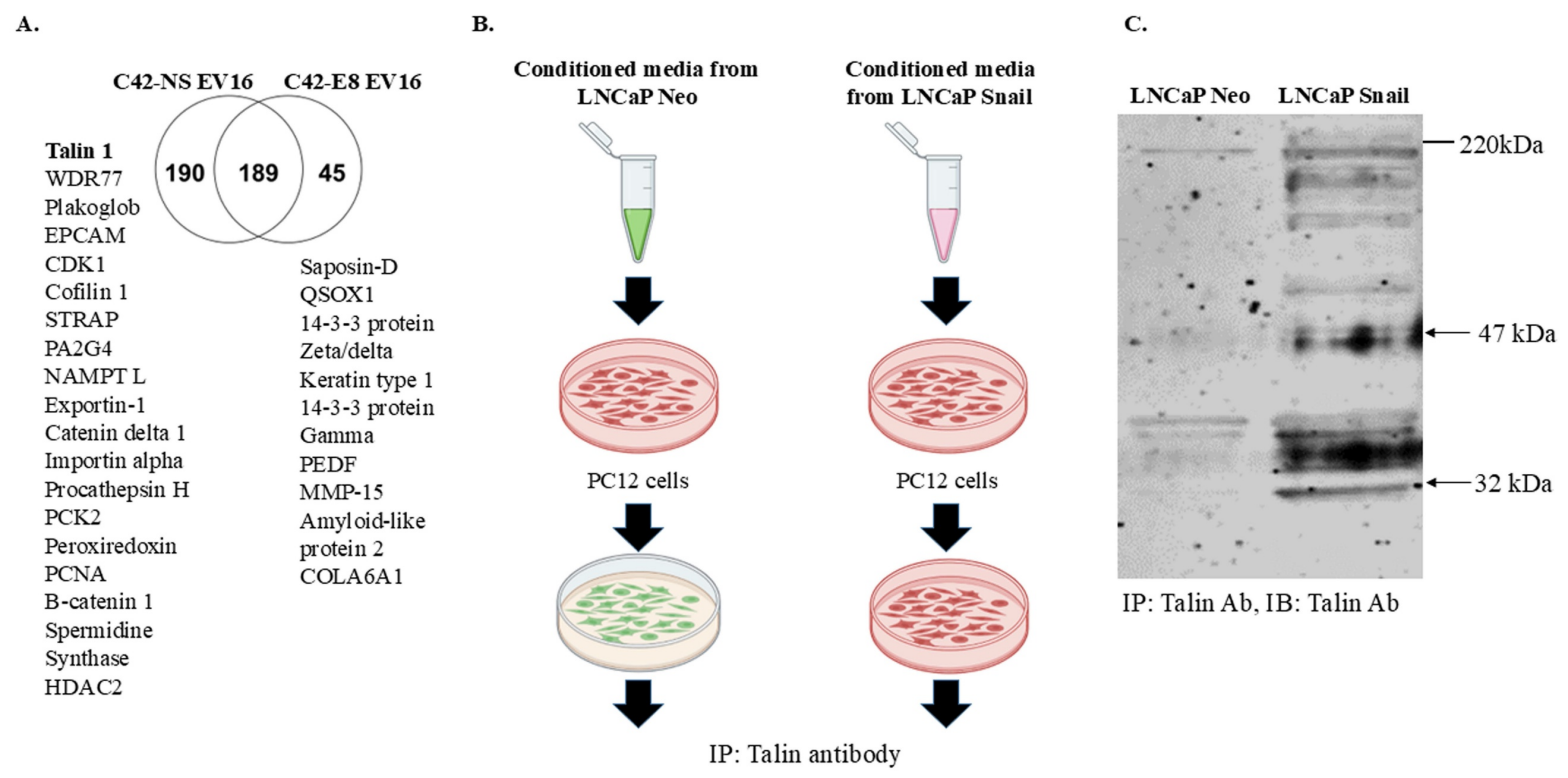
** Proteomic profile content of extracellular vesicles indicates Talin1 as a possible mediator of cancer cell-nerve interactions.** (A) Extracellular vesicle populations were enriched from cell supernatant of C4-2NS (control) or C4-2 E8 (Snail knockdown) by differential ultracentrifugation at 300, 3000, and 10,000xg. The vesicles were then subjected to an in-solution digestion with trypsin, and peptides were analyzed with a Q-exactive Orbitrap mass spectrometer. A Venn diagram is shown depicting common and specific proteomes found in C4-2 NS as compared to C4-2 E8. (B) Conditioned media from LNCaP Neo or LNCaP Snail cells (Snail overexpression) were added to enriched plasma membrane fractions from PC12 cells. (C) Samples were subjected to IP with anti-Talin1 antibody followed by western blot with anti-Talin1 antibody. The result is representative of at least 3 independent experiments.

Past and current studies indicate that extracellular vesicles and proteins secreted from cancer cells are vital in cancer-nerve crosstalk in diverse malignant tumors 40. Therefore, we decided to understand the factors stimulating neurite outgrowth in Snail-expressing cells. We first conducted a proteomic study by isolating extracellular vesicles using differential ultracentrifugation, followed by trypsin digestion and analysis via mass spectrometry. We used C42-NS/E8 cell model since the absence of Snail can significantly increase the analytical value of the potentially altered proteins released by cancer cells in a Snail-dependent manner. The Venn diagram in Figure 3A indicates that Talin1 is one of the proteins found in extracellular vesicles from C4-2 NS (Snail-expressing) cells and is absent in C4-2 E8 Snail knockdown cells. Talin1 has been reported to promote neurite outgrowth in nerve cells 24.

To further investigate the role of Talin1, we performed Talin1 antibody Immunoprecipitation (IP) with membrane fractions of PC12 cells cultured with conditioned medium collected from LNCaP-Neo or LNCaP-Snail cells (Figure 3B). The results showed a significant increase in Talin1 protein, particularly the 47 kDa N-terminal head domain, the 220 kDa C-terminal rod domain and a 32 kDa isoform in the membranes of PC12 cells cultured in CM from Snail-overexpressing LNCaP cells (Figure 3C). Together, these results indicate that the Snail-Talin1 axis can be a causal mechanism underlying the reciprocal interactions between tumors and the tumor-associated nervous system, elevating the therapeutic value of Snail in solid tumors.

### Whole cell lysates and exosomes from PCa and BCa cells contain Talin1

**Figure 4:**
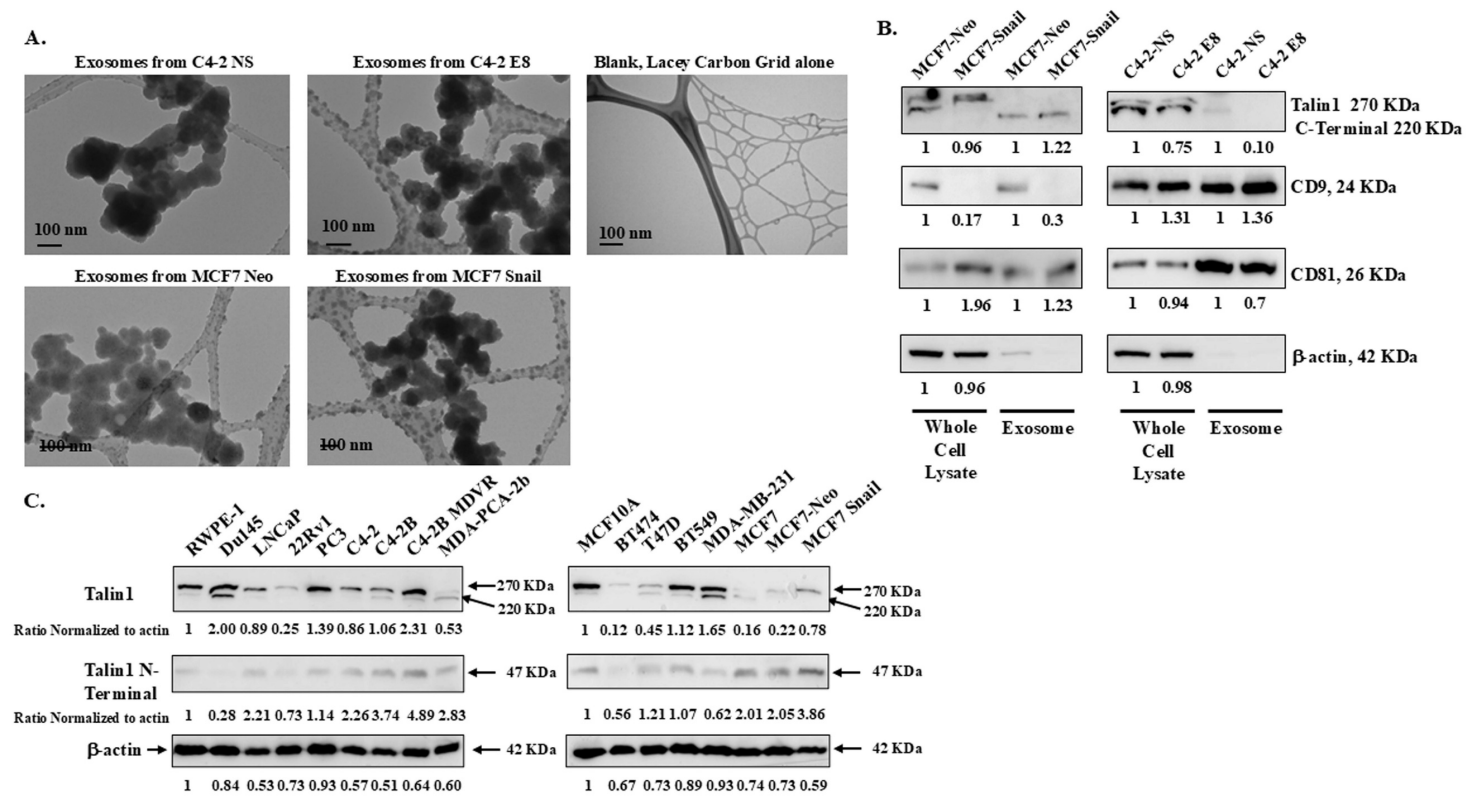
** Exosomes purified from conditioned media of BCa and PCa cells contain Talin1** (A) Transmission electron microscopy was performed to verify isolated exosomes. (B) Talin1, exosome marker proteins CD9 and CD81 were detected in the whole cell lysate or the purified exosome fractions. (C) Talin1 expression in a panel of BCa and PCa cancer cells was analyzed by Western blot analysis. Quantification of Talin1, CD9, and CD8 was performed using ImageJ software and normalized to β-Actin, which was utilized as a loading control. Results are representative of triplicate experiments performed independently.

Exosomes, which are extracellular vesicles secreted into the extracellular space, contain unique proteins and RNAs that promote cell-cell communication 25, 26. We isolated exosomes from CM collected from C4-2 non-silencing (NS), C4-2 Snail knockdown (E8), MCF7-Neo, and MCF7-Snail overexpression cells. These exosomes were imaged under a transmission electron microscope (TEM), and their sizes were found to be slightly smaller than 100 nm, confirming that they are within the size attributed to exosomes (Figure 4A). Additionally, western blot analysis confirmed that two common exosomal markers, tetraspanins CD9 and CD81, were detected in the isolated exosomes (Figure 4B).

We also examined the Talin1 protein levels in both whole cell lysates and isolated exosomes from MCF-7 Neo/Snail or C4-2 NS/E8. In whole cell lysates harvested from exosome-free media, the 270 kDa full-length and the 220 kDa isoform Talin1 were detected (Figure 4B). However, the 220 kDa proteolyzed Talin1 C-terminal rod domains are predominant in the isolated exosomes (Figure 4B). Exosomes from MCF7-Snail contained a roughly 20% increased amount of the 220 kDa Talin1 compared to those from MCF7-Neo (Figure 4B). At the same time, exosomes from C4-2 E8 with Snail knockdown contained less Talin1 compared to C4-2 NS cells (Figure 4B).

We also examine Talin1 expression among various prostate and breast cell lines grown in complete medium to determine whether there is a meaningful correlation between Snail expression and Talin1 proteins in these cancer cells. Interestingly, PCa cells expressing low levels of Snail protein exhibited reduced Talin1 expression. Among the PCa cell lines, 22Rv1 and MDA-PCA-2b, which showed the lowest Snail expression, also expressed the lowest levels of Talin1 (Figure 4C). MDA-PCA-2b cells predominantly expressed the 220 KDa C-terminal domain (Figure 4C). Among the breast cancer cell lines, the highest expression of the 270 kDa full-length Talin1 was observed in MCF10A, MDA-MB-231, and BT549, followed by MCF7-Snail cells (Figure 4C). Talin1 220 KDa C-terminal domain was highly expressed in MDA-MB-231 cells, with lower levels detected in T47D and MCF7 cells (Figure 4C).

Interestingly, a 47 KDa N-terminal fragment was observed in both PCa and BCa cell lines, but with no apparent pattern (Figure 4C). Results in Figure 4 suggest Talin1 full-length and proteolytic isoforms can be observed in PCa and BCa whole cell lysates and also within exosomes.

### Exosomes from Snail-expressing cells promote increased neurite outgrowth of human Neural Progenitor Cells (NPCs), which can be antagonized by mH4 Talin1 inhibitor

To investigate the interaction between human cancer cells and nerve cells more effectively using human neurons, we utilized human Neural Progenitor Cells (NPCs) for a neurite outgrowth assay with the addition of purified exosomes or CM in the presence/absence of 280 µg/ml mH4 (Talin1 inhibitor). Since we observed proteolytic products of Talin1 within CM and exosomes of cancer cells, we collaborated with Kaytee Bio, which was developing inhibitors against the short form of Talin1 for Rheumatoid Arthritis (RA). Normally, the N-terminal of Talin-1 is cleaved into a 47-kDa fragment by calpain-1 (Figure S1A). However, it was found that when Rheumatoid Arthritis (RA) patient plasma is added, this 47-kDa fragment is further cleaved into 32-kDa and 15-kDa fragments (Figure S1B). When a proprietary short form Talin1 protease inhibitor (mH4) is added, the shorter isoforms are no longer observed, while they are still observed with a control F3 control protease inhibitor (Figure S1B). This suggests that mH4 can inhibit Talin1 cleavage. Subsequently, we cultured NPCs with either medium (regular media used to culture NPCs) or RPMI-1640 with 10% exosome-depleted FBS (Exo-D medium) as negative controls. NGF-beta was utilized as a positive control. Other NPC cells were cultured with either CM collected from cancer cells or Exo-D medium with exosomes purified from cancer cells.

**Figure 5:**
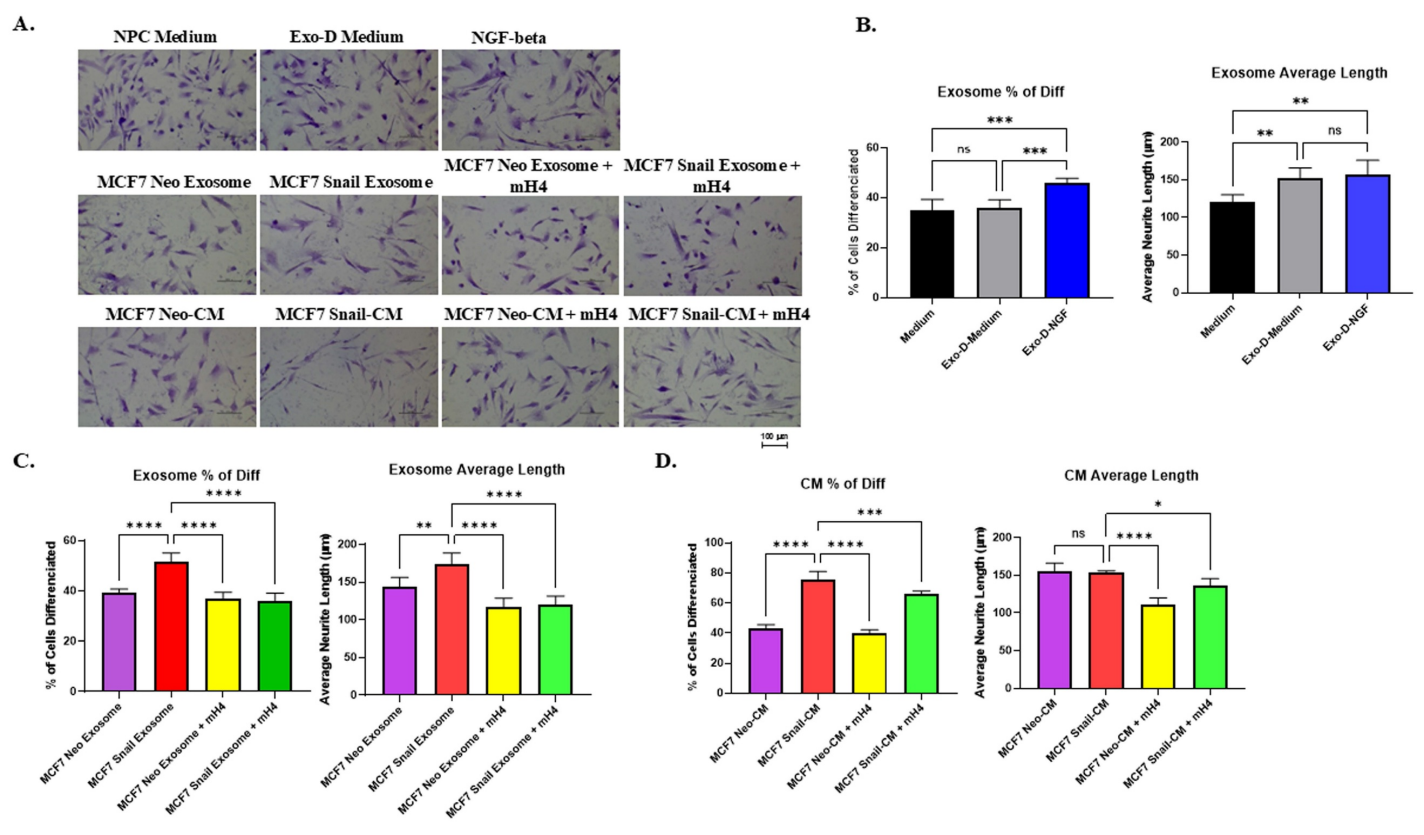
** Human Neural Progenitor Cells (NPCs) display increased neurite outgrowth when cultured with exosomes and CM from MCF-7 Snail cells compared to MCF-7 Neo.** MCF-7 Neo or MCF-7 Snail was cultured in exosome-depleted medium (Exo-D medium) for 3 days, and conditioned medium (CM) was collected, or exosomes were isolated from the CM. These were subsequently cultured with NPCs for 2 days. (A) Images of NPC after incubation in NPC regular medium, Exo-D medium, exosomes or CM with or without Talin1 inhibitor mH4 were captured with a Nikon Eclipse TE2000-S Inverted Fluorescence Microscope. (B) The percentage of NPCs with neurite outgrowth and average neuron length was measured utilizing Image J Fiji Just software in NPCs cultured with NPC medium, Exo-D medium, and Exo-D medium plus NGF-beta as a positive control. The percentage of NPCs with neurite outgrowth and average neuron length of NPCs cultured with (C) exosomes isolated from MCF7-Neo and MCF7-Snail CM or (D) CM from MCF7-Neo and MCF7-Snail was analyzed using ImageJ Fiji Just software. Statistical analyses were done using GraphPad Prism software (one-way ANOVA, Šídák's multiple comparisons test, ****p< 0.0001, ***p< 0.001, **p < 0.01, *p < 0.05). Bars represent the SD of the mean. Results are representative of 3 experiments performed independently.

Figure 5A shows that NGF-beta resulted in a moderate increase in the percentage of outgrowth NPCs, indicating increased neurite outgrowth. After quantification with Image J Fiji Just software, an increased percentage of cells with neurite outgrowth and average neurite length were observed in the NPCs cultured with Exo-D-NGF beta compared to NPC medium and Exo-D medium (Figure 5B). There was a significant increase in the percentage of outgrowth neurite due to MCF7-Snail exosomes and CM compared to Neo control, which was antagonized by mH4 Talin1 inhibitor (Figure 5C and 5D). Moreover, exosomes from Snail-expressing MCF7 cells significantly increased average neurite outgrowth length in the NPCs compared to MCF7-Neo, more so than CM, and this was antagonized by mH4 (Figures 5C and 5D).

**Figure 6:**
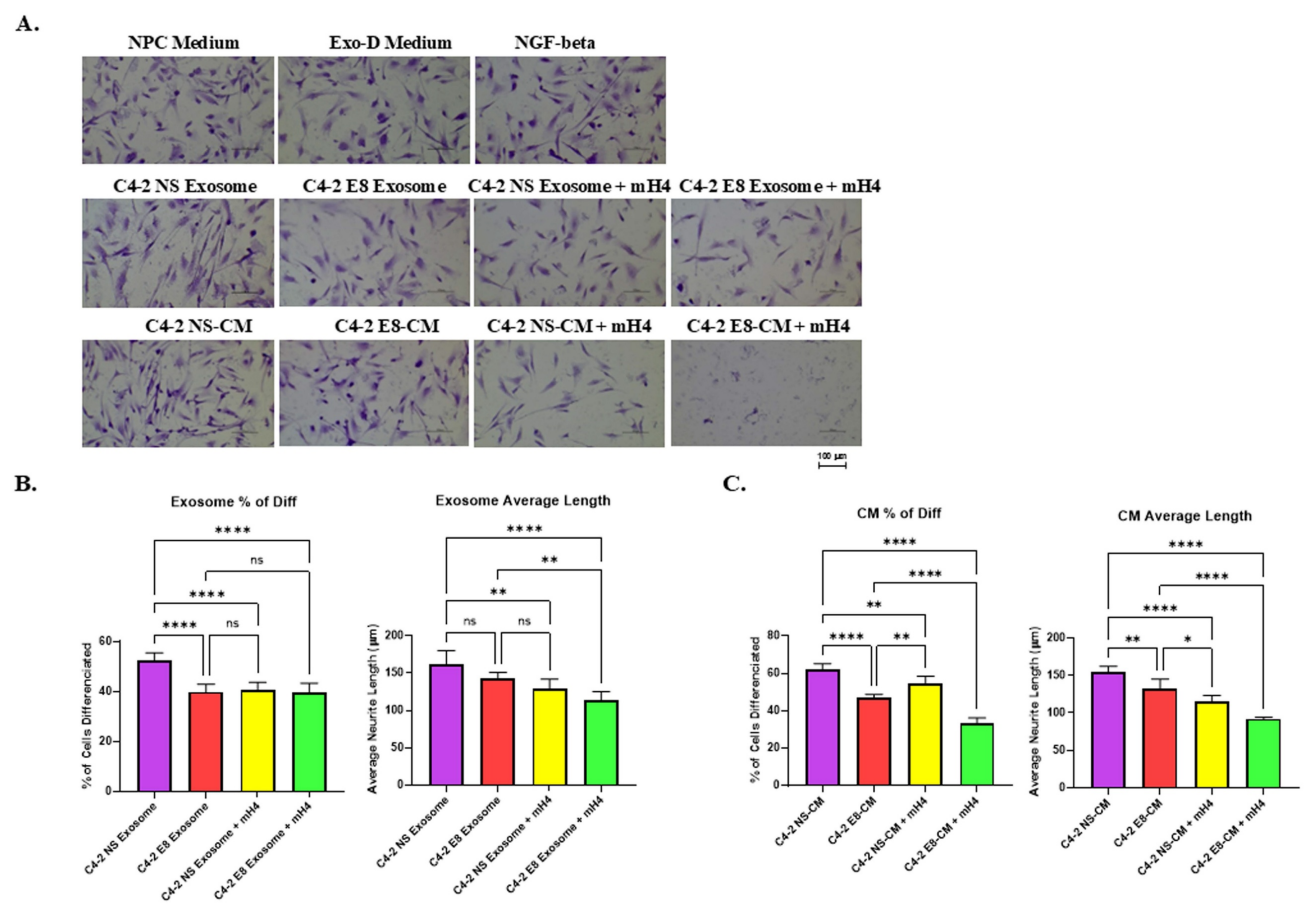
** Human neural progenitor cells (NPCs) display reduced neurite outgrowth with exosomes and CM from C4-2 Snail knockdown (C4-2 E8) cells compared to C4-2 Non-silencing (NS) cells.** C4-2 NS or C4-2 E8 was cultured in exosome-depleted medium (Exo-D medium) for 3 days, and conditioned medium (CM) was collected, or exosomes were isolated from the CM. These were subsequently cultured with NPCs for 2 days. (A) Images of NPC after incubation in NPC regular medium, Exo-D medium, exosomes, or CM with or without Talin1 inhibitor mH4 were captured with a Nikon Eclipse TE2000-S Inverted Fluorescence Microscope. The percentage of NPCs with neurite outgrowth and average neuron length of NPCs cultured with (B) exosomes isolated from MCF7-Neo and MCF7-Snail CM or (C) CM from MCF7-Neo and MCF7-Snail was analyzed using ImageJ Fiji Just software. Statistical analyses were done using GraphPad Prism software (one-way ANOVA, Šídák's multiple comparisons test, ****p< 0.0001, **p < 0.01, *p < 0.05). Bars represent the SD of the mean. Results are representative of 3 experiments performed independently.

A similar experiment was performed with NPCs cultured with CM from C4-2-NS and C4-2-E8, or the purified exosomes, and cell morphology was visualized (Figure 6A). Exosomes or CM from PCa cells with Snail knockdown (C4-2-E8) resulted in a reduced percentage of cells with neurite outgrowth compared to the C4-2-NS counterparts (Figures 6B and 6C). However, there was a trend toward reduced average neurite length that was not significant in NPCs cultured with exosomes C4-2 E8 compared to C4-2 NS (Figure 6B), while average neurite length was significantly reduced by Snail knockdown (Figure 6C). The mH4 Talin 1 inhibitor reduced the percentage of cells with neurite outgrowth as well as the average neurite length in all the conditions except for C4-2 E8 exosomes (Figure 6B and 6C). Overall, exosomes and CM from Snail-expressing BCa and PCa cells can promote neurite outgrowth in NPCs, which can be antagonized by Talin1 inhibitor, mH4.

### AKT signaling in PC12 cells is activated by Snail-expressing cancer cells and suppressed by Snail knockdown cells or Talin1 inhibitor

**Figure 7:**
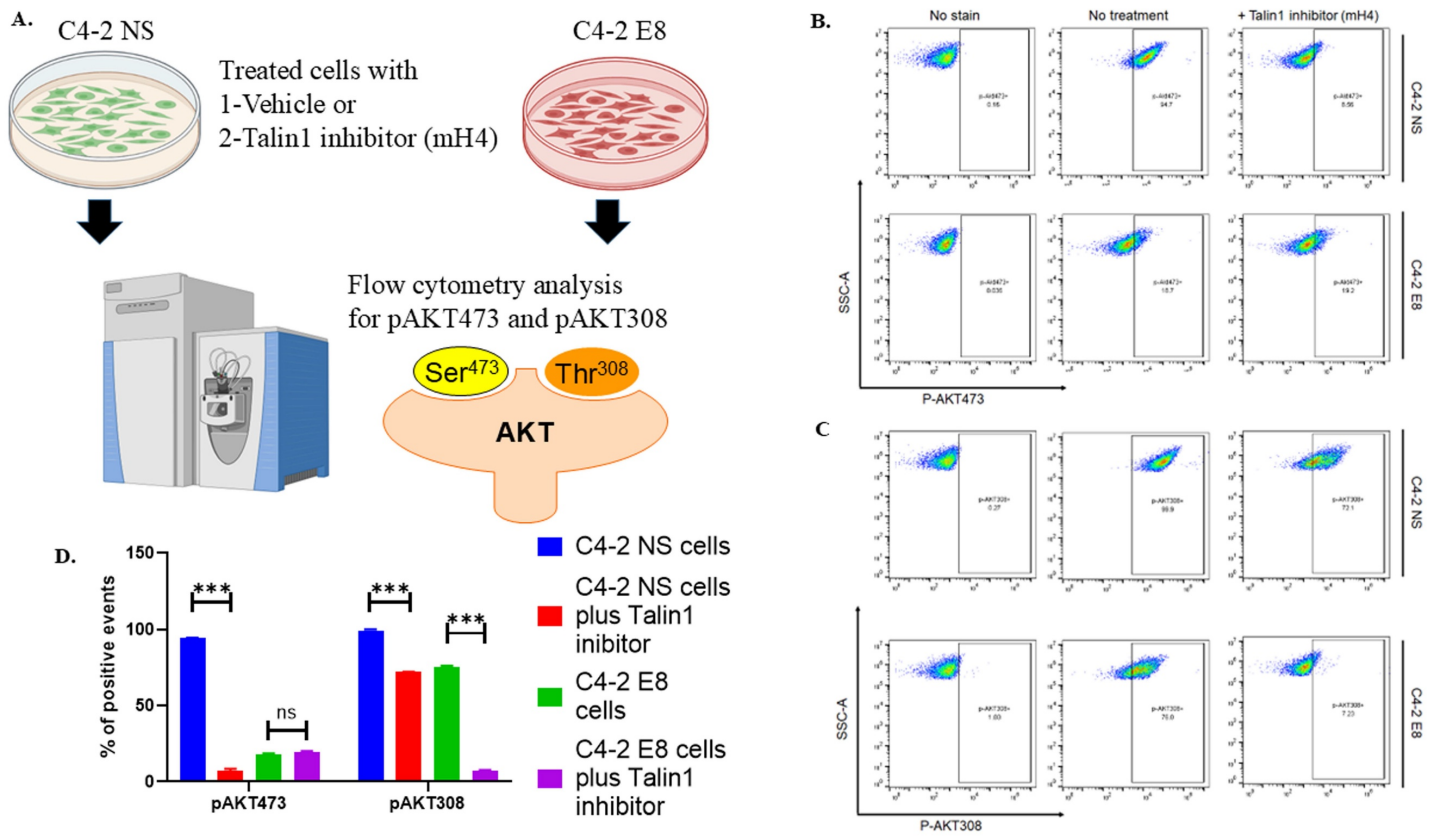
** Talin inhibitor mH4 impairs activation of the PI3-AKT pathway in PC12 cells cultured with conditioned media from cancer cells.** (A) C4-2 NS control cells with non-silencing shRNA and C4-2 cells with stable shRNA Snail knockdown (C4-2 E8) were treated with PBS or mH4 inhibitor for 48 hours. Conditioned medium was subsequently collected and added to PC12 nerve cells for 24 hours. PC12 nerve cells were stained with (B) anti-p-AKT (S473) and (C) anti-pAKT308 Alexa Fluor 546 antibodies, followed by flow cytometry analysis. (D) Statistical analyses were done using GraphPad Prism software (one-way ANOVA, Šídák's multiple comparisons test, ***p< 0.001). Bars represent the SD of the mean. Results represent triplicate experiments.

We then proceeded to examine the potential pathways involved in the Snail/Talin1-stimulated cancer/nerve cell interaction. We collected CM from PBS control or mH4-treated C4-2-NS and C4-2-E8 cells. PC12 cells cultured with these CM were collected and analyzed using antibody-associated flow cytometry to study the activation of the PI3K-AKT pathway. The levels of both p-AKT (S473) and pAKT308 were significantly reduced by mH4 in the presence of Snail in C4-2 NS cells (Figure 7, blue column versus red column). Interestingly, the level of pAKT308 decreased in the presence of mH4 in Snail-silenced cells (Figure 7, green versus purple column). To achieve full activation, AKT must be phosphorylated at T308 by extracellular signals and at S473 intracellularly by mTORC2, respectively. Thus, cancer cell interaction with nerve cells involves AKT signaling.

## Discussion

Interactions between cancer cells and nerves have been shown to promote both BCa and PCa cell progression and metastasis 4, 7. Snail, a potent regulator of the epithelial-mesenchymal transition (EMT), a process integral to metastasis, is inversely associated with cancer patient survival 9. Our previous work demonstrated that, beyond its role in EMT, Snail expression in PCa cells enhances interactions with nerve cells by increasing adhesion, migration towards neurons, and neurite outgrowth 13. In the present study, we extended these findings to BCa cells and further explored the mechanism(s) of action. We found that MCF7 BCa cells overexpressing Snail promote increased neurite outgrowth compared to Neo control. Mechanistically, Talin1, an integrin-binding protein crucial for focal adhesions, mediates the induction of neuronal cell differentiation by Snail-expressing cancer cells. Our novel findings indicate that Talin1 is found in extracellular vesicles, particularly exosomes of Snail-expressing PCa and BCa cells. Almost all the Talin1 detected in isolated exosomes consists of the 220 kDa C-terminal rod and 47 kDa head domains, which are proteolytic isoforms of the full-length 270 kDa Talin1. The 220 kDa domain is known to bind to β integrins, vinculin, and F-actin and is stimulated by the 47 kDa head domain, which interacts with FAK and β integrins 18, 19. Intriguingly, the 220 kDa C-terminal rod domain is secreted into exosomes. The presence of the activated 47 kDa N-terminal and 220 kDa C-terminal Talin1 in the interacting PC12 nerve cellular membrane, as well as the presence of the 220 kDa C-terminal in the secreted exosomes, suggests that Talin1 may be responsible for the neurite outgrowth induced by high Snail expression. Our study is supported by a previous finding that overexpression of Talin1 in PC12 cells promotes neurite outgrowth 41. Interestingly, in this study, full-length Talin1 increased neurite length, while the Talin1 head domain increased the percentage of neurite-bearing cells, and the Talin1 rod domain increased integrin activation. Another study showed that Talin1 is a mechanosensor-mediated structural force required for axonal growth and regeneration 42.

Talin1 expression in PCa showed a distinct positive association with Snail expression, whereas no clear association was observed in BCa cells. Notably, the two BCa cell lines with the highest Talin1 levels, MDA-MB-231 and BT 549, are triple-negative breast cancer (TNBC) cell lines, implying a potential link between Talin1 and TNBC. Supporting this notion, Zhang *et al.* (2022) reported that blocking the interaction between Talin1 and integrin β1 suppresses TNBC progression 23. The lower levels of 220 kDa rod domain and the higher levels of the 47 kDa head domain in MCF7-Snail cells suggest that the rod domain may be selectively secreted through exosomes into the surrounding microenvironments, where it could function as a mitogenic factor promoting nerve cell differentiation. Analysis of Talin1 isoforms distribution in the exosomes and whole-cell extract revealed a clear dichotomy, where the majority of full-length Talin1 resides in cell lysates, while the 220 kDa rod domain is predominates in purified exosomes.

mH4, a proprietary Talin1 inhibitor that inhibits cleavage of Talin1, inhibited Snail-mediated neurite outgrowth in our study. This differs from previous findings, where calpain cleavage of Talin1 inhibits Src-dependent axonal growth in *Xenopus laevis* 43, 44. The difference between these studies suggests differential regulation by Talin1 in different species. Perhaps that would need further clarification in the future.

The PI3K-AKT pathway is a key regulator of growth and survival signaling. Both p-AKT (S473) and p-AKT (T308) were reduced in the PC12 nerve cells cultured with CM from C4-2 Snail knockdown cells, compared to C4-2 NS. Additionally, mH4, significantly decreased p-AKT S473 and T308 phosphorylation of PC12 cells cultured with C4-2 NS CM. With CM from C4-2 E8 that already had low p-AKT, mH4 further inhibited p-AKT T308 but not p-AKT S473. These findings suggest that Snail expression in cancer cells and Talin1 cleavage may regulate nerve behavior through PI3K signaling, a mechanism that requires further investigation. Of note, the combination of mH4 plus C4-2 NS/E8 exosomes or CM showed some toxicity by reducing cell numbers, particularly with C4-2 E8 CM plus mH4, which was not observed with MCF7 Neo/Snail, suggesting cancer or cell-line specific differences. We will further investigate the suitable dose range of mH4 in both cancer and NPC cells that can efficiently reduce the neurite outgrowth without affecting normal cell growth in PCa cells to reduce toxicity. Further experiments will also determine the effect of mH4 on cancer cells to examine the possibility of utilizing mH4 to target both cancer cells that express Talin1 cleavage products and NPCs.

In summary, our work identified that Talin1 secreted in exosomes from high Snail-expressing cancer cells may stimulate neurite outgrowth on neuronal cells via the AKT signaling pathway. Additionally, the proprietary Talin1 inhibitor mH4, which prevents cleavage of Talin1, was found to effectively reduce the stimulated neurite outgrowth, suggesting that Talin1 cleavage products are important for neurite outgrowth. These findings suggest that mH4 may hold promise as a potential therapeutic agent for preventing PCa and BCa progression through antagonizing cancer-nerve cell interactions.

## Supplementary Material

Supplementary materials and methods, figures and table.

## Abbreviations

PCa: prostate cancer
BCa: breast cancer
NPC: neural progenitor cells
NGF: nerve growth factor
TNBC: triple-negative breast cancer
DRG: dorsal root ganglia
EMT: epithelial-mesenchymal transition
TCGA-PRAD: The Cancer Genome Atlas Prostate Adenocarcinoma
CtsH: cathepsin H
FAK: focal adhesion kinase
RNA: ribonucleic acid
DNA: deoxyribonucleic acid
kDa: kilodalton
CM: conditioned media
CD9: cluster of differentiation 9
CD81: cluster of differentiation 81
mTORC2: mechanistic target of rapamycin complex 2
BCA: bicinchoninic acid
HRP: horseradish peroxidase
IP: immunoprecipitation
SDS/PAGE: sodium dodecyl sulfate-polyacrylamide gel electrophoresis
PBS: phosphate-buffered saline
TEM: transmission electron microscopy
SD: standard deviation
