# Supplementary material for: Talin1 Mediates Tumor-Nerve Interactions in Prostate and Breast Cancer Cells
Source: J Cancer. 2026 Jan 8;17(2):359–71. doi: 10.7150/jca.127292 (PMC12825424; doi:10.7150/jca.127292)
Supplement: Supplementary file 1 — Supplementary materials and methods, figures and table. [file jcav17p0359s1.pdf]

SUPPLEMENTARY MATERIAL

Supplementary Material and Methods

Reagents

Talin1 inhibitor, mH4 and F3 protease inhibitor control, was obtained from Dr. Kensei Tsuzaka, Kaytee Bio, Co. & Ltd., Chiba, Japan. mH4 was dissolved in DMSO to a stock solution of 14 mg/ml. Rheumatoid arthritis (RA) patient plasma was obtained from Tokyo Dental College Ichikawa General Hospital, Chiba, Japan. GST Bulk kit, pGEX vector, anti-GST antibody were purchased from Cytiva, Marlborough, MA. BL21(DE3) competent cells is from ThermoFisher, Waltham, MA.

Purification of GST N-Terminal 47 KDa Talin1

The N-terminal 47 KDa Talin1 cDNA was cloned into a pGEX vector and expressed as a GST fusion protein in E. coli. strain BL21. Purification was performed using the GST Bulk kit (Cytiva, 100 Results Way, Marlborough, MA).

Cleavage of GST-Talin N-Terminal 47 KDa Talin1 with plasma of Rheumatoid Arthritis (RA) patient

GST-Talin1 (47 KDa) was mixed with 0, 30, 90, and 270μg, respectively of F3 (control protease inhibitor) or mH4, and incubated with 45μL of plasma from a RA patient for 1h at room temp. The mixture was resolved in a 15% of SDS-PAGE gel followed by Electro-Blue staining. 47 KDa and 32 KDa Talin1 fragments were confirmed in Western blot using anti-GST antibody (Data not shown).

Table S1. The properties of PCa and BCa cells used in this research.

**Prostate Cancer Cell lines**					
**Name**	**Collected from**	**Origin**	**Doubling time**	**Age**	**Race**
**RWPE-1**	normal adult human prostate cells were transfected with a single copy of the human papilloma virus 18 (HPV-18)	NPEC in peripheral zone	**120 h** [a]	54	White
**Du145**	Central nervous system (Brain)	Brain metastasis	**34 h** [a]	69	White
**LNCaP**	Lymph node	lymph node metastatic	**28-60 h** [a]	50	White
**22Rv1**	Prostate	CWR22R xenograft derivative	**35-40 h** [a]	NA	White
**PC3**	Bone	lumbar vertebral metastasis	**33 h** [a]	62	White
**C4-2**	Bone	vertebral metastasis LNCaP cell xenograft to mouse	**~48 h** [a]	50	White
**MDA-PCA-2b**	Bone	from an African-American male	**42-73 h** [a]	63	Black
**Breast Cancer Cell lines**					
**Name**	Collected from	Disease	**Doubling time**	Age	Race
**MCF10A**	Breast; Mammary gland	benign proliferative breast tissue and spontaneously immortalized without defined factors	**~48 hour** [b]	36	NA
**BT-474**	Breast; Duct; Mammary gland	Ductal Carcinoma	**3~4 day** [d]	60	White
**T47D**	Breast; Mammary gland	Carcinoma; Ductal	**~37 hour** [c]	54	NA
**BT-549**	Breast; Mammary gland	Carcinoma; Ductal	**3.7 day** [d]	72	White
**MDA-MB-231**	Breast; Mammary gland	Adenocarcinoma	**~20 hour** [c]	51	White
**MCF7**	Breast; Mammary gland	Adenocarcinoma	**~24 hour** [b]	69	White

References: a. PMID: 26146646; b. PMID: 17445777; c. PMID: 32061169; d. Cellosaurus: https://www.cellosaurus.org/

A. **B.**

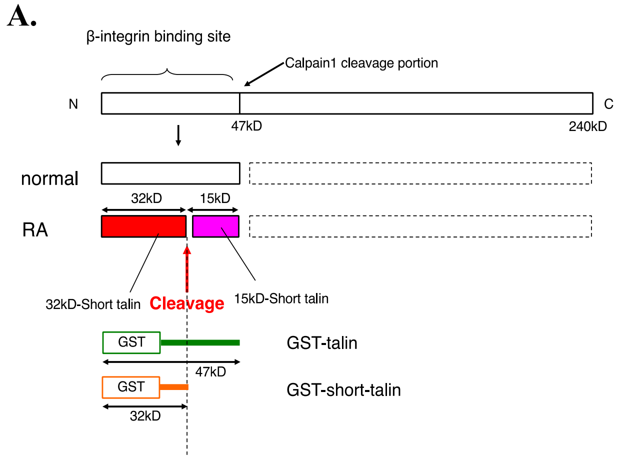

Figure S1. Development of a proprietary Talin1 inhibitor mH4

(A) Talin1 can be cleaved into 220 kDa and 47 kDa fragments by Calpain 1. The 47 kDa N-terminal Talin1 was cloned into pGEX vector for the studying the further proteolysis in Rheumatoid Arthritis (RA) patients. The 47 kDa can be further cleaved into 32 kDa short Talin. (B) GST-short Talin (32kDa) was observed when incubated with 270μg of F3 but was not observed with the same amount of mH4, suggesting mH4 has inhibited the protease that cleaved the 47 kDa into short Talin.

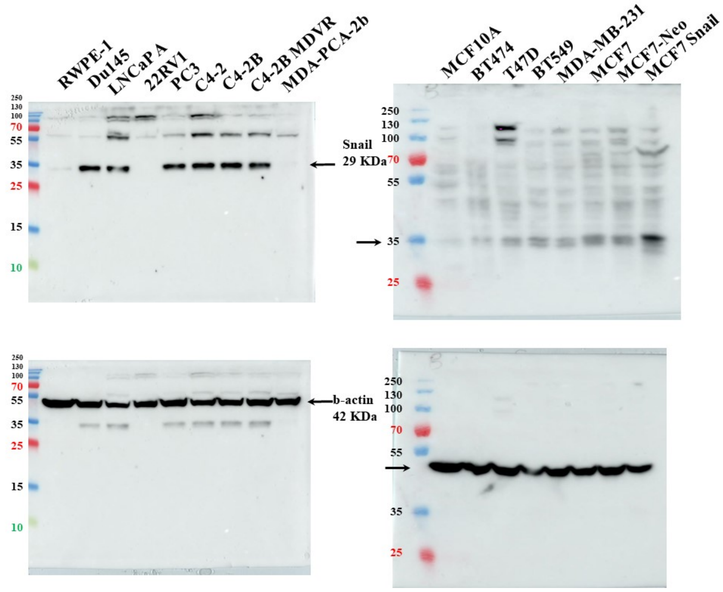

Figure S2. Full blot images for Figure 1

Images were captured using the Amersham™ Imager 680. All images were taken prior to saturation of the target bands.

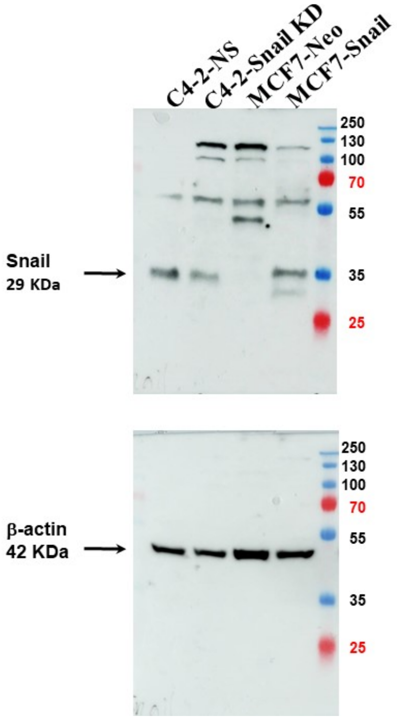

Figure S3. Full blot images for Figure 2E

Images were captured using the Amersham™ Imager 680 and analyzed with ImageQuant TL software for intensity quantification when needed. All images were taken prior to saturation of the target bands.

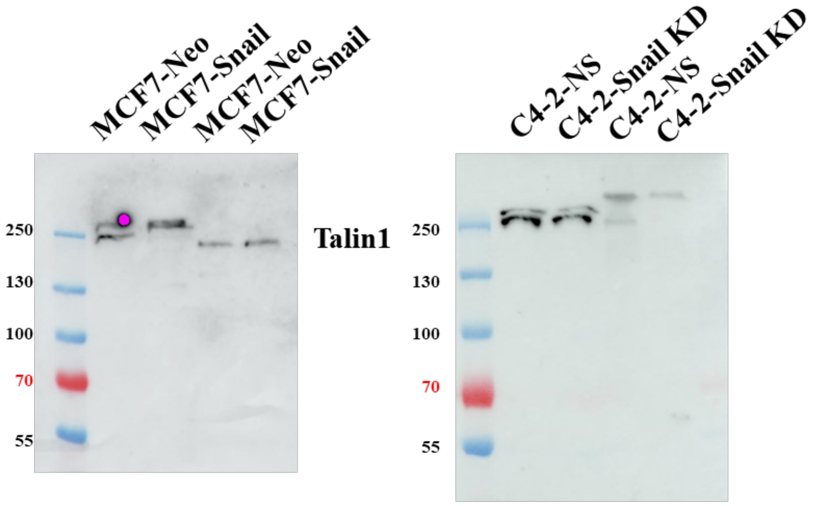

Figure S4. Full blot images for Figure 4B, talin1

Images were captured using the Amersham™ Imager 680 and analyzed with ImageQuant TL software for intensity quantification when needed. All images were taken prior to saturation of the target bands.

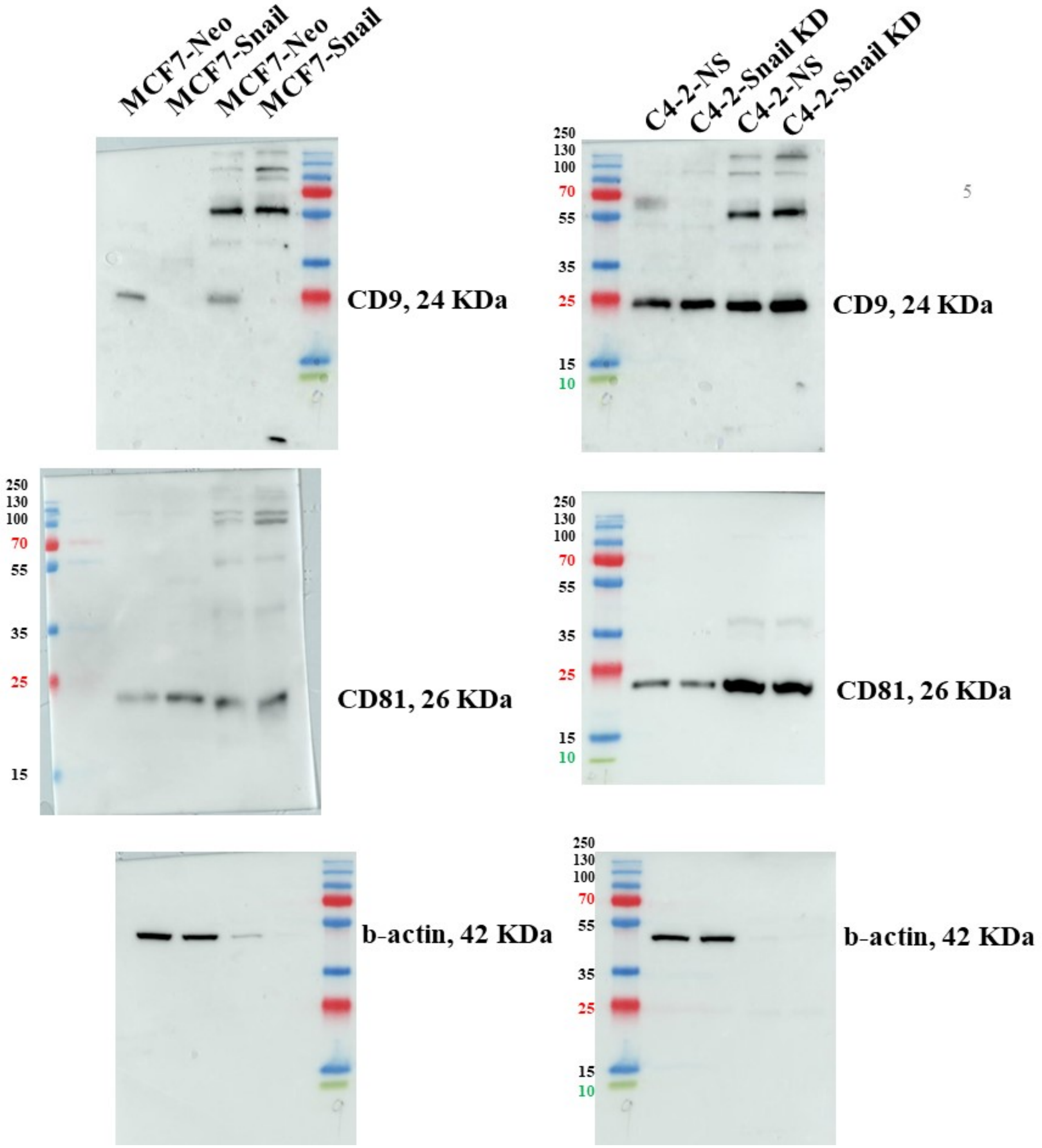

Figure S5. Full blot images for Figure 4B, CD9, CD81, and beta actin

Images were captured using the Amersham™ Imager 680 and analyzed with ImageQuant TL software for intensity quantification when needed. All images were taken prior to saturation of the target bands.

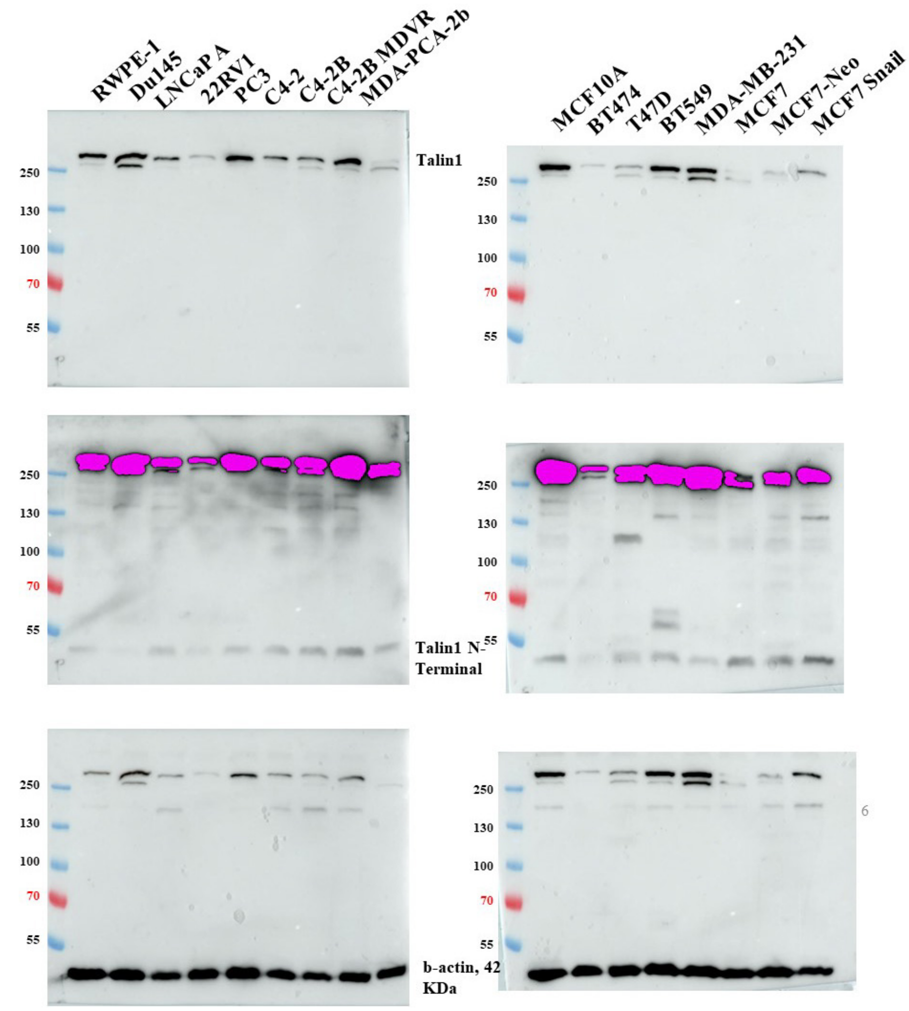

Figure S6. Full blot images for Figure 4C

Images were captured using the Amersham™ Imager 680 and analyzed with ImageQuant TL software for intensity quantification when needed. All images were taken prior to saturation of the target bands. In Figure 4C, the middle images were intentionally overexposed to enhance detection of the 47 kDa Talin1 band, resulting in overexposure of the 270 and 220 kDa Talin1 bands.
